# Telemedicine and Plastic Surgery: A Pilot Study

**DOI:** 10.1155/2015/187505

**Published:** 2015-11-02

**Authors:** Denis Souto Valente, Luciano Silveira Eifler, Lauro Aita Carvalho, Gustavo Azambuja Pereira Filho, Vinicius Weissheimer Ribeiro, Alexandre Vontobel Padoin

**Affiliations:** ^1^Graduate Program in Medicine and Health Sciences, PUCRS School of Medicine (FAMED), Avenida Ipiranga 6681, 90619-900 Porto Alegre, RS, Brazil; ^2^Mae de Deus Health System, Rua Soledade 569, 90470-340 Porto Alegre, RS, Brazil

## Abstract

*Background*. Telemedicine can be defined as the use of electronic media for transmission of information and medical data from one site to another. The objective of this study is to demonstrate an experience of telemedicine in plastic surgery. *Methods*. 32 plastic surgeons received a link with password for real-time streaming of a surgery. At the end of the procedure, the surgeons attending the procedure by the Internet answered five questions. The results were analyzed with descriptive statistics. *Results*. 27 plastic surgeons attended the online procedure in real-time. 96.3% considered the access to the website as good or excellent and 3.7% considered it bad. 14.8% reported that the transmission was bad and 85.2% considered the quality of transmission as good or excellent. 96.3% classified the live broadcasting as a good or excellent learning experience and 3.7% considered it a bad experience. 92.6% reported feeling able to perform this surgery after watching the demo and 7.4% did not feel able. 100% of participants said they would like to participate in other surgical demonstrations over the Internet. *Conclusion*. We conclude that the use of telemedicine can provide more access to education and medical research, for plastic surgeons looking for medical education from distant regions.

## 1. Introduction

Telemedicine can be defined as the use of electronic media for transmission of information and medical data from one site to another. It is a vague term which cover a wide range of topics, all concerning the delivery of health care at a distance, encompassing diagnosis and treatment of patients, education of staff, patients, and the general public, and administrative activities, such as collecting public health data, as well as research. All of these may be assisted by judicious use of telemedicine. The exchange of medical information over distances by electronic means is an emerging area [1, 2].

The main advantage of telemedicine is that it can improve access to health care, often by reducing the need to travel or by increasing the speed with which a specialist opinion can be obtained. The classical telemedicine programs have mainly used store-and-forward methods, although there has been some limited use of real-time video. Its applications range from data collection for diagnostic purposes to telesurgery [3]. The objective of this study is to demonstrate the initial experience of the authors in the application of telemedicine in plastic surgery.

## 2. Materials and Methods

The demonstration of a malar fat pad removal surgery for aesthetic purposes is reported. After obtaining informed consent from the patient, and with the support of the Hospital Telemedicine Service, a demonstrative surgery was scheduled. 32 members of the Brazilian Society of Plastic Surgery who wanted to see how this surgery was done were warned by e-mail 12 days in advance and received a link with password for real-time streaming. This study is in accordance with the 2000 Edinburgh, Scotland Revision of the Declaration of Helsinki, applicable ICH guidelines, and Guidelines on Research Practice.

**Figure 1:**
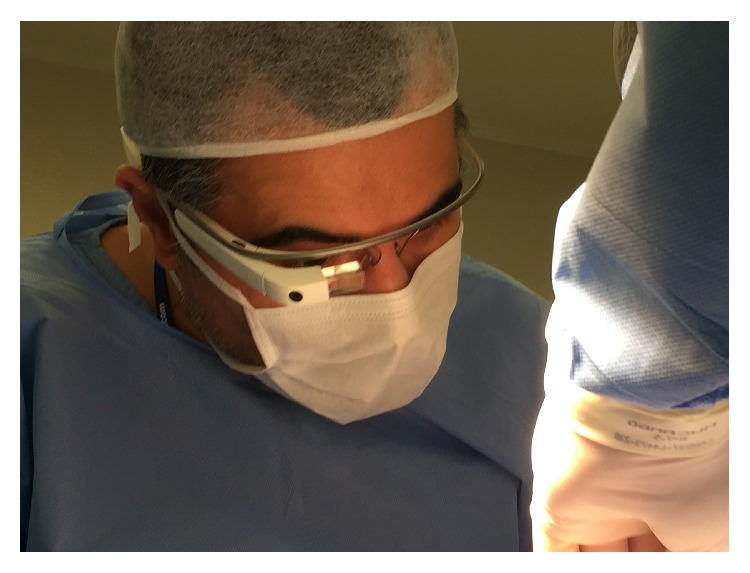
The plastic surgeon wearing the Glass.

**Figure 2:**
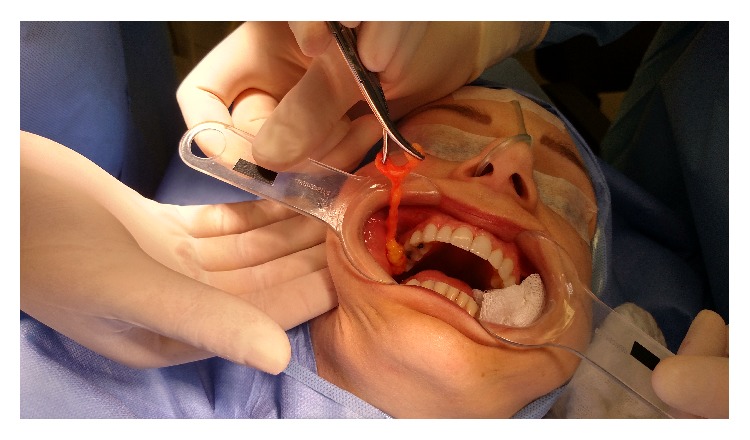
An image captured by the equipment.

On the day of surgery, the plastic surgeon wore a Google Glass (Google Inc., California, USA) and performed the surgery with transmission via Internet in real time. The live broadcast was interactive and the observers were able to interact remotely with the operating surgeon.  Figure 1 shows the plastic surgeon wearing the Glass, and Figure 2 shows an image captured by the equipment. At the end of the procedure, the surgeons who attended the procedure by the Internet answered five questions, two related to transmission and three related to the learning goals in this broadcast. At the end of data collection, the results were presented and analyzed with descriptive statistics.

## 3. Results

Among the 32 invited plastic surgeons, 27 attended the online procedure in real time. 96.3% considered the access to the website as good or excellent and 3.7% considered it bad. 14.8% reported that the transmission was bad and 85.2% considered the quality of transmission as good or excellent. 96.3% classified the live broadcasting as a good or excellent learning experience and 3.7% considered it a bad learning experience. 92.6% reported feeling able to perform this surgery after watching the demo and 7.4% did not feel able. 100% of participants said they would like to participate in other surgical demonstrations over the Internet.

## 4. Discussion

Telemedicine allows a reduction in consultation time, training, and mentoring among different geographic areas without the expensive mobilization of experts. Telemedicine modalities can range from elementary transmission of digital texts or images with medical context, through live interactive videoconference, to complex procedures as performing surgery in a remote location via robotic tools. E-health and telehealth refer to the delivery of remote clinical and nonclinical services using technology. The transmission of still images, patient consultations by videoconferencing, patient interactive portals, continuing medical education, patient-focused wireless applications, remote monitoring of vital signs, and nursing call centers, among other utilizations, are all considered as telemedicine. Technological advances in picture, audio, and video tools for Internet sharing, wireless broadband availability, and the evolution of handheld devices allowed patients to access medical services without the need to travel long distances shortening the gap among medical facilities and persons needing medical care. The first generation telemedicine systems were “point-to-point” models over landlines, confining health care providers to fixed workstations within hospitals. The involvement of the World Wide Web heralded the second generation of telemedicine, allowing consultations to be conducted from anywhere at any time [1, 4].

Plastic surgery can benefit from telemedicine more than other medical specialties because the clinical visual assessment is the basis of the diagnosis of traumatic injuries, burns, wounds, and the arrangement of transoperative care. Usually, plastic surgery does not depend on laboratorial or imaging tests. The advanced broadcasting technology has been convenient to plastic surgeons as a diagnostic gadget and can simplify the clinical assessment from a distance [5].

An important benefit of telemedicine is to provide skilled medical care. Complex wounds and severe traumatic injuries can be evaluated remotely to afford the appropriate venue for transfer and treatment. Persons with chronic wounds usually have several mobility limitations; telemedicine can be useful in these patients allowing plastic surgeon evaluation, as well as wound monitoring and care, generating logistic and financial benefits. Patients who do not have access to specialist care for geographical reasons can benefit from telemedicine consultations without the need to travel long distances, thus generating savings from work or school absence prevention, and the suffering of elongated waiting periods to achieve specialist evaluations. Telemedicine has been used in military facilities and also by humanitarian purposes to provide international multidisciplinary care to war victims living in remote areas who do not have access to specialty care [6, 7].

There is a debate about the ethical aspect of telemedicine. The Federal Council of Medicine in Brazil defines and regulates the provision of services through telemedicine as practice of medicine through the use of interactive methodologies, audiovisual communication, and data, for care, education, and research in health. The services provided through telemedicine should have the infrastructure and appropriate technology, to comply with the technical standards relevant to custody, handling, data transmission, confidentiality, privacy, and assurance of medical secrecy [8].

By analyzing the data obtained in our work, we see that the vast majority of study participants found the experience useful and all of which accompanied the surgical procedure wanted to participate in new dynamics using the same technology. This opens an important path for worldwide plastic surgery information exchange among plastic surgeons. The American Society of Plastic Surgeons believes that telemedicine is one of the future fields of research in this specialty [9]. In our paper, there is not a conflict of interests, because the only quoted equipment (Google Glass) is no longer manufactured worldwide. This study's main limitations include the small sample size, the short study duration, and the observational design.

This technology allows surgeons to track one procedure to their displacement distance decreasing costs and time and exposing the patient to lower risk of infection if there were more people in the operating room. In addition, the images may be stored in Cloud enabling better low-cost medical documentation. Data support Google Glass positive impact on health care delivery, clinical training, medical documentation, and patient safety. Concerns exist regarding patient confidentiality, technical issues, and limited software [10].

Telemedicine is now widely used in surgery from performing operations to teaching and can be divided into three main components: telesurgery, telementoring, and teleconsultation. Developments across these fields have led to remarkable achievements such as intercontinental telesurgery and telementoring [11]. In plastic surgery, it is now being performed for burn management, real-time video consultation, cleft care, microsurgery monitoring, hand surgery, and wound management [1, 2, 4–7, 12, 13].

Although telemedicine technologies are considered promising, most telemedicine applications have failed to survive beyond the funded research phase to be embedded as methods of routine health care delivery [14, 15]. An important issue in our study is the interaction possibility. If there was not an interactive component, there would not be an educational benefit of the live broadcast studied by just watching a recorded video through Google Glass. As the observers were permitted to interact with the surgeon, we found a use of telemedicine that has been a well-described tool for surgical education and the observers feedback was an interesting analysis. Currently, medical education has not taken advantage of the advances in telemedicine. Information about this mode of medical delivery remains absent from the medical school curriculum. The next generations of physicians, who will be the users of this new transformative system, have little foundation in telemedicine. This is something that needs to change.

## 5. Conclusion

The use of telemedicine can provide more access to education and medical research, for plastic surgeons looking for medical education from distant regions. This translational technology can positively impact health care delivery, medical documentation, surgical training, and patient safety.
